# Establishing a method of HPLC involving precolumn derivatization by 2,2′‐dithiobis (5‐nitropyridine) to determine the sulfites in shrimps in comparison with ion chromatography

**DOI:** 10.1002/fsn3.1060

**Published:** 2019-05-15

**Authors:** Kai Yang, Cheng Zhou, Zhenhuan Yang, Lan Yu, Ming Cai, Changqing Wu, Peilong Sun

**Affiliations:** ^1^ Department of Food Science and Technology Zhejiang University of Technology Hangzhou China; ^2^ Department of Animal and Food Sciences University of Delaware Newark, Delaware; ^3^ Zhoushan Yueyang Food Co., Ltd Zhoushan China

**Keywords:** determination, HPLC, precolumn derivatization, shrimps, sulfites

## Abstract

Although sulfites are widely used in shrimp processing, the contents of residual sulfite need to be strictly controlled due to their potential toxicity. In this paper, a novel method was developed for determination of the free and total sulfites in shrimps. Major procedures of the method includes separation of free and total sulfites with ultrasound‐assisted extraction and pH adjustment for 20 min, then a precolumn derivatization was conducted by 2,2′‐Dithiobis (5‐nitropyridine) and verified by LC‐MS, and finally HPLC coupled with an ultraviolet (UV) detector was carried out. Results indicated that the UV absorption wavelength shifted from 213 (sulfites) to 320 nm (new disulfide compounds), significantly reducing the interference of natural occurring compounds and solvents in the matrix. The standard curves exhibited a good linear range of 3.2–51.2 mg/L (*R* ^2^ = 0.9996). The limit of detection (LOD) and limit of quantification (LOQ) were 0.3 and 1.0 mg/L, respectively. The contents of free and total sulfite in frozen shrimps were 26.58 ± 0.48 and 31.44 ± 0.83 mg/kg calculated by SO_2_, respectively. These were similar (*p* > 0.05) to the data obtained by the method of ion chromatography. In conclusion, the new developed method has been proved to be a reliable and economic method for effective determination of free and total sulfites in the shrimps, and the method could be expanded in determination of the sulfites in other food products.

## 1 INTRODUCTION

Shrimp has been valued as delicious and nutritional food in daily diets. But it is easi to be deteriorated or blackened during their catching and transportation without any special treatments (Takeungwongtrakul, Benjakul, & Hkittikun, 2012). Including cold storage, sulfites were usually added as preservatives into shrimp during transportation and storage (Lou et al., 2017). Recent evidences also suggested that sulfites had the function of preventing food spoilage and oxidation, inhibiting microbial growth, and controlling enzymatic and nonenzymatic reactions (McWeeny, Biltcliffe, Powell, & Spark, 2010; Sayavedrasoto & Montgomery, 2010). The major forms existed as free and bound sulfites, when they were added to the foodstuff. The sum of free and bound sulfites was referred to as total sulfites, and reversibly bound sulfites could be released by extraction with an appropriate pH (McLeod & Davey, 2007).

Sulfites are widely used in food processing and hard to be replaced, because they are economic and easy for application (Zhang et al., 2014). Despite of these remarkable advantages, sulfites should be applied with strict limitation due to their potential toxicity (da Costa Machado Matos Carvalho et al., 2011). People may suffer from asthmatic and allergic reactions if they ingested foods containing large amounts of sulfites, especially in free (or reversibly bound) sulfite form (Moseholm, Taudorf, & Frøsig, 2010; Reno, Brooks, & Ameredes, 2015). Besides, long‐term exposure to sulfites can cause neurotoxicity and even damage to the reproductive system (Kencebay et al., 2013; Marshall, Reist, Jenner, & Halliwell, 1999). Many countries and international organizations have laid strict restrictions on the contents of sulfite in different foodstuffs. Codex Alimentarius Commission (CAC) sets maximum sulfite limitation in raw and cooked shrimp as 100 and 30 mg/kg, respectively. Besides, the U.S. Food and Drug Administration (FDA) and European Food Safety Authority (EFSA) had stipulated that if the residues of sulfites in food exceeds 10 mg/kg (EFSA, 2016), it must be labeled on the packaging (EFSA, 2016; Ruiz‐Capillas & Jiménez‐Colmenero, 2009).

Irreversibly bound sulfites are hard to be detected by common analytical techniques, because of their extraordinary stable forms. A variety of methods have been used in the detection of sulfites in dried fruits and vegetables (Ni, Tang, Liu, Shen, & Mo, 2015), beverages (Theisen, Hansch, Kothe, Leist, & Galensa, 2010), wines (Aberl & Coelhan, 2013; Guarda et al., 2016), and aquatic products, including chemical titration (Daniels et al., 1992; Moinierwilliams, 1927), spectrophotometry (Filik, 2012), electrochemical methods (Wang & Xu, 2014), and ion chromatography (Zhong, Zhu, Luo, Huang, & Wu, 2012). However, almost all the methods have showed some disadvantages, including high cost, longtime processing (including the cumbersome sample preparation and subsequent analysis), poor environmental stability, or uncommon instruments. Therefore, it is urgent to develop a more convenient and reliable method for determination of sulfites in foodstuff.

High‐performance liquid chromatography (HPLC) is widely used nowadays. HPLC coupled with multi‐wavelength spectrophotometer was used to analyze the free and total sulfites in foodstuffs (Lim et al., 2014; Pizzoferrato, Di, & Quattrucci, 1998). HPLC instruments, sometimes combined with preseparation treatments, were used to analyze the sulfites in fresh sausages, shrimps, grapes, dehydrated fruits and vegetables, and the results were similar to those obtained by traditional Monier‐Williams distillation method (Ni et al., 2015; Pizzoferrato, Quattrucci, & Di, 1990).

Three main pretreatment methods were available for the determination of sulfites in foods by HPLC: (a) direct determination of the sulfite concentration of the sample solution; (b) oxidation of sulfite to sulfate, and (c) sulfite derivatization (Jackowetz & Orduña, 2013; McFeeters & Barish, 2003). The ultraviolet absorption wavelength of sulfites is 213 nm, at which a large number of natural compounds (such as protein and amino acids) and organic solvents can absorb and cause disturbances, resulting in decreased accuracy. Compared with other methods, the derivatization method possessed obvious superiority with lower detection limits and less interference.

Sulfite ions can react with many organic disulfides to displace thiol anions and form organic thiosulfates, commonly referred as “Bunte” salts (Field, 1977). The reaction is according to Equation 1.

(1) $$\text{RSSR}+{\text{SO}}_{3}^{2-}⇌{\text{RSSO}}_{3}^{-}+{\text{RS}}^{-}$$

**Figure 1:**
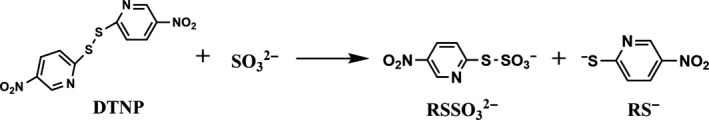
The derivatization reaction between DTNP and SO_3_^2−^. DTNP, 2,2′‐Dithiobis (5‐nitropyridine)

The disulfide (RSSR) reacts quantitatively with sulfite ions (SO_3_^2−^) to generate new sulfite‐containing dithio compounds (RSSO_3_^−^) and thiol (RS^−^). In almost all cases, the disulfides can be fully reacted with sulfite ions in a ratio of 1:1 stoichiometry, and this principle was often used for the detection of disulfides (Li & Zhao, 2006). In addition, nitro‐containing disulfides were more sensitive to this type of reaction, and traces amounts of sulfite ions can react quantitatively with nitro‐containing aromatic disulfides, making it detectable by UV spectrophotometer (Humphrey, Ward, & Hinze, 1970). Certain disulfides, particularly of 2,2′‐Dithiobis (5‐nitropyridine) (DTNP), were commonly used to determine the thiol in various biochemical samples. The reaction process of DTNP and SO_3_^2−^ was shown in Figure 1.

Herein, we aimed to separately extract the free and total sulfites from shrimps and then quantify by precolumn DTNP derivatization HPLC (PD‐HPLC). Both detection limit and recovery rate were evaluated, and this proposed method has been also verified and compared with ion chromatography.

## 2 MATERIALS AND METHODS

### 2.1 Reagents and materials

Sodium sulfite standard solution (Na_2_SO_3_, 0.01 mol/L), propyl alcohol, acetic acid, sodium acetate, hydrochloric acid, and sodium hydroxide were all purchased from Aladdin Reagent Co., Ltd, and 2,2′‐Dithiobis(5‐nitropyridine) (DTNP, 96%) was purchased from Sigma‐Aldrich. Methanol and acetonitrile of HPLC grade reagents were purchased from Tianjin Siyou Fine Chemicals Co., Ltd. Ultrapure water was self‐prepared by Direct‐Q5 water machine (Merck Millipore Inc.). pH of the extraction process was measured by a PHS‐3F pH meter (Shanghai Yidian Scientific Instrument Co., Ltd). Frozen shrimps were provided by Zhoushan Yueyang Food Co., Ltd, and stored at −18°C.

### 2.2 Preparation of samples

Frozen shrimps were delivered to the laboratory by an ice incubator in two hours with the temperature kept below −10°C. Once delivered, it was stored in a refrigerator at −18°C for further use. Prior to the analysis, the shrimps were homogenized using a meat grinder (JYS‐A960 Joyoung), then weighed accurately (10,0000 g), put into a 250‐ml Erlenmeyer flasks, and sealed. 50 ml of propyl alcohol (1%, v/v) solution was added to stabilize sulfite and prevented oxidation.

The method of separation of sulfites was based on the previous method (Wang & Xu, 2014) with slight modifications. The extraction solution was adjusted to pH 8.0 for free sulfite extraction and pH 12.0 for total sulfite extraction (Rita, Maria, & Eduardo, 2009), using 0.5 mol/L of sodium hydroxide, then extracted with the assistance of ultrasonic leaching for 15 min, and centrifuged (Bechman Allegra model) at 7104 g for 15 min at 4°C. The supernatants were transferred to a volumetric flask and then diluted with ultrapure water to be 100 ml for HPLC measurements.

### 2.3 Derivatization process

8.0 ml of ultrapure water, 0.5 ml of 0.2 mol/L acetic acid–sodium acetate buffer, 1.0 ml of the above prepared standard or sample solution, and 0.5 ml of 5.0 mmol/L DTNP solution (dissolved in acetonitrile) were added in turn to a 20 ml test tube and mixed thoroughly for 30 s at 25°C. The mix was filtered through a 0.22 μm membrane (Millipore) for HPLC analysis.

### 2.4 HPLC conditions

High‐performance liquid chromatography separation was performed using a Waters 1525 chromatography systems (Waters Inc.), with a Waters 2487 dual‐channel UV–visible detector at wavelength of 320 nm. The injection (20 μl) was automatic using a W2707 autosampler, and a C_18_ column (5 μm; 4.6 × 250 mm internal diameter) from Waters was used. Column temperature was kept at 30°C in a column oven, and samples were held in the autosampler tray at 20°C prior to the injection. The mobile phase were as follows: 0.05 mol/L acetic acid–sodium acetate solution (A) and pure acetonitrile (B). The flow rate used was 1.0 ml/min. The gradient elution procedures were as follows: 90% A, 0–1 min; 90%–66% A, 1–10 min; 66%–45% A, 10–25 min; 45%–0% A, 25–35 min; and 90%A, 35–45 min.

Intraday precision determination included the following: the retention time and the RSD value of the peak area of seven continuous injections; interday precision: the retention time and the RSD value of five injections in 3 days.

### 2.5 LC‐MS analysis

LC‐MS analysis was carried out on a Waters 2695 coupled with MS spectrometry (Thermo Fisher LCQ™ Deca XP plus) and using a Waters C_18_ column (5 μm; 4.6 × 250 mm). The mobile phase was 5 mmol/L ammonium acetate (A) and pure acetonitrile (B), and the elution procedure was the same as HPLC. Negative electron spray ionization mass spectrometry (ESI‐MS) was used to analyze the chemical structure of sulfite derivatives.

### 2.6 Calibration curves and recoveries of sulfites

Calibration curves were done with 0, 0.05, 0.1, 0.2, 0.4, and 0.8 mmol/L of sulfite solutions prepared by dilution from a 0.01 mol/L stock solution. The stock solution was formulated with 10 mmol/L of propyl alcohol to inhibit oxidation.

Recoveries of sulfites were evaluated by the addition of sodium sulfites (5.0, 20, and 50 mg/kg, calculated as SO_2_) to the cover solution of shrimp homogenates at pH 8.0 or pH 12.0. Triplicate samples were prepared from the separated solution and analyzed for both free and total sulfites before and after adding of sodium sulfites. The sulfite concentration was calculated using the standard curves.

### 2.7 The PD‐HPLC method compared with the IC method

ICS‐1100 ion chromatography system equipped with a conductivity detector (Thermo Fisher Scientific Inc) and a Dionex IonPac AS9‐HC Column (4 mm × 50 mm) were used to determine sulfites. Parallel samples of sulfite were extracted in the same method as PD‐HPLC and determined using an IC method according to Industry Standard of China with minor modification (SN/T 2918–2011, 2011). After centrifugation, the sulfite containing supernatant was purified by a solid phase extraction column activated (5.0 ml of methanol and 5.0 ml of deionized water) and then filtered through a 0.22 μm membrane. The eluent was a mixture of 8.0 mmol/L of Na_2_CO_3_ and 2.5 mmol/L of NaOH, at a flow rate of 1.0 ml/min, and the injection volume was 25 μl. The results are expressed in terms of SO_2_ and compared with the data of the PD‐HPLC method.

### 2.8 Data analysis

All analyses were triplicated, and the results were expressed as mean ± *SD*. SPSS version 19.0 (IBM, 2018) was used to conduct all statistical analyses. A *t* test was used for comparison between two means, and a one‐way analysis of variance (ANOVA) was used for comparison of more than two means; a *p* value of less than 0.05 was assumed to be statistically significant.

## 3 RESULTS AND DISCUSSION

### 3.1 UV absorption spectrum

**Figure 2:**
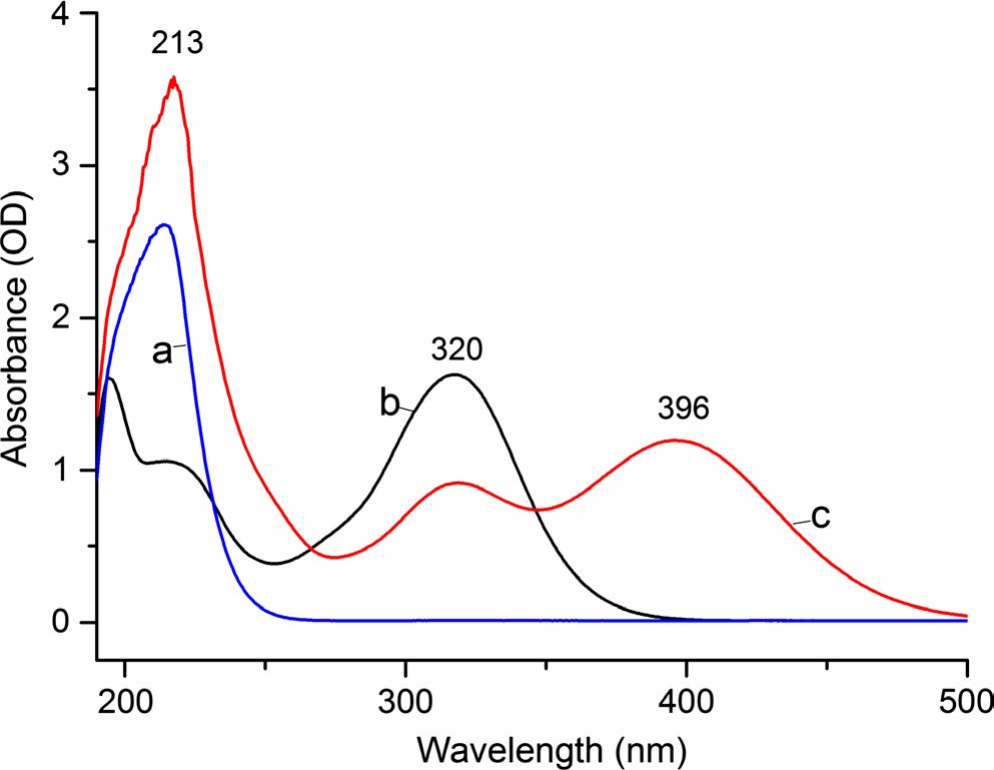
The UV and visible absorption spectra of SO_3_^2−^ before and after DTNP derivatization. (a) 2 × 10^−3^mol/L SO_3_^2−^, (b) 2 × 10^−4^ mol/L DTNP, and (c) 2 × 10^−3^ mol/L SO3^2−^ derivatized by 2 × 10^−4^ mol/L DTNP. DTNP, 2,2′‐Dithiobis (5‐nitropyridine)

The absorption spectra of SO_3_^2−^, DTNP, and the reaction solution with a slight excess of sulfites were shown in Figure 2. As seen from the curves, the maximum absorption of standard SO_3_^2−^ and DTNP (representation of the disulfide) solution was at 213 and 320 nm, respectively. The reaction solution had absorption peaks at 213 (SO_3_^2−^), 320 (disulfide compound, RSSO_3_^−^), and emerging 396 nm (RS^−^, 5‐Nitro‐2‐mercaptopyridine, the derivatives of mercapto compounds). These results were consistent with the reports of similar compounds (Humphrey et al., 1970; McFeeters & Barish, 2003). As the reaction solution containing excess of sulfite ion, and the UV absorption spectra showed a reduced absorption peak around 320 nm compared with original DTNP, it was speculated that there might be some new reaction products, such as the disulfide compounds containing sulfites. This speculation will be verified by LC‐MS.

### 3.2 Chromatograph analysis

**Figure 3:**
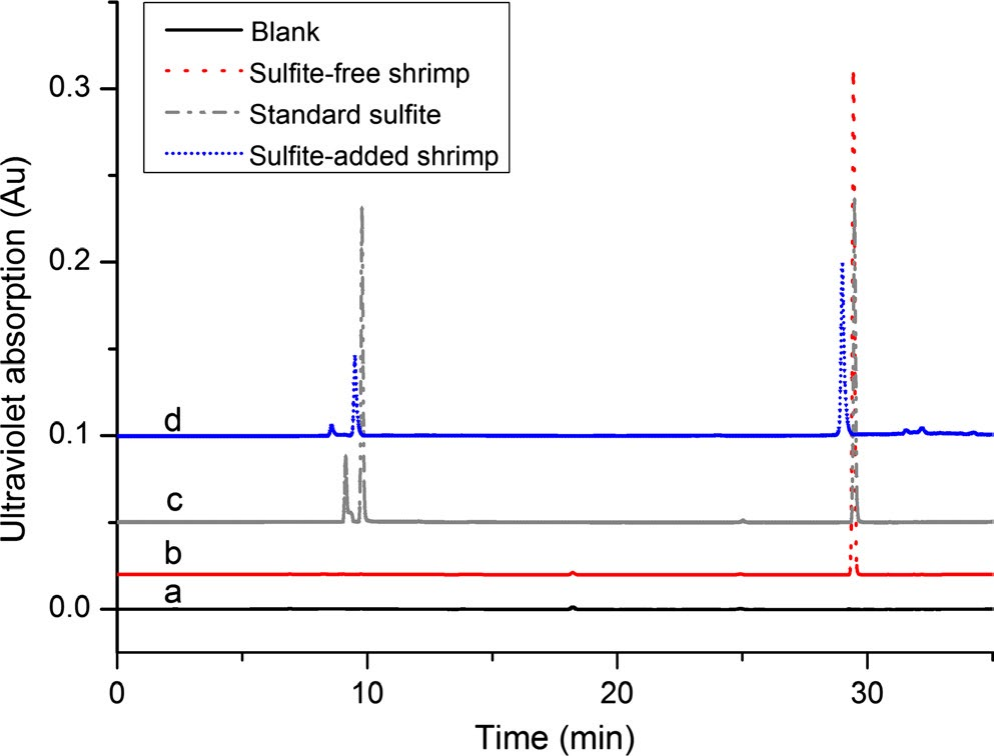
The chromatogram of different components. (a) pure solvent (blank); (b) shrimp sample without added sulfite (sulfite‐free shrimp); (c) standard sulfite; (d) shrimp sample added with sulfite (sulfite‐added shrimp)

**Figure 4:**
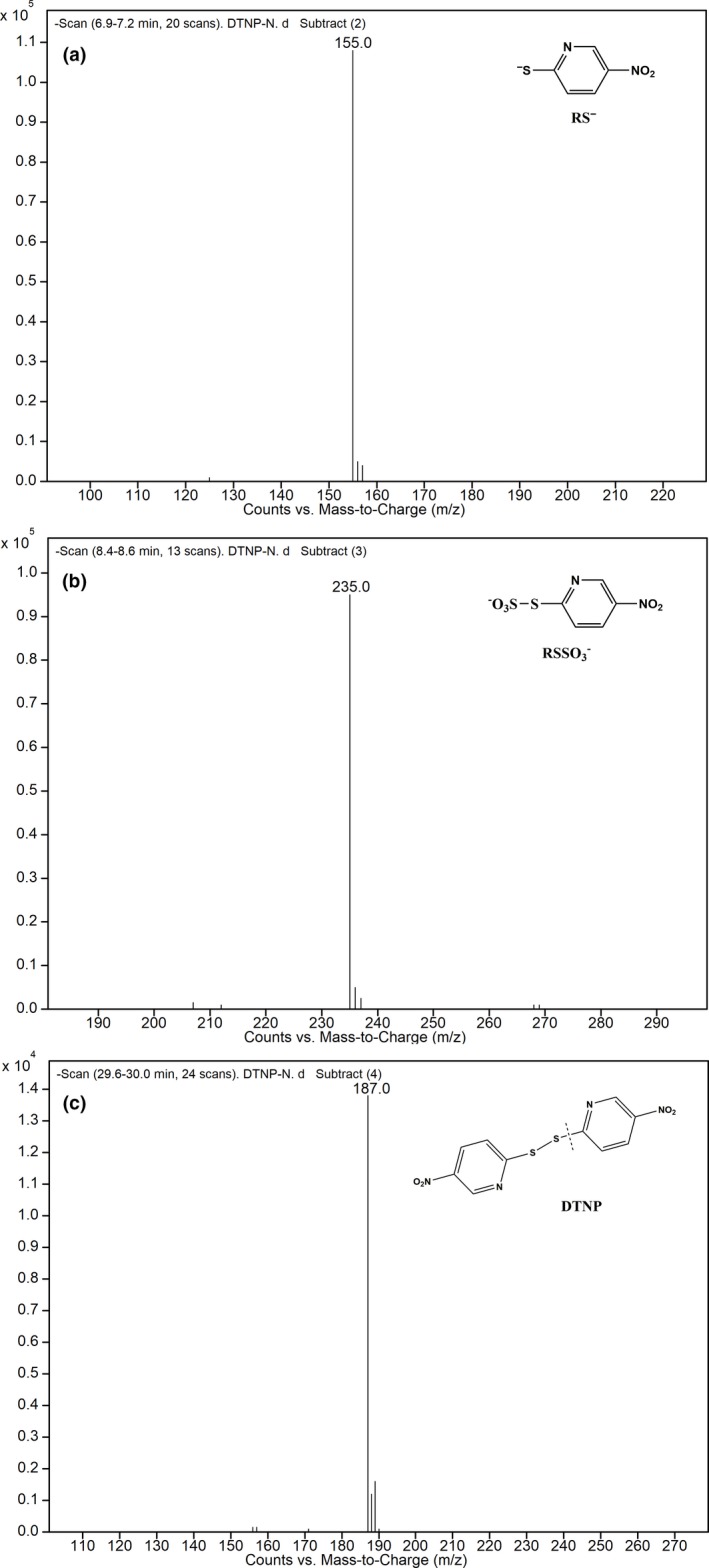
The MS profiles of sulfites derivative with DTNP. DTNP, 2,2′‐Dithiobis (5‐nitropyridine)

Figure 2 displayed that the absorption wavelengths of the sulfites and DTNP derivative products were shifted from 213 to 320 nm, significantly increasing the selective detection of sulfites. This was also verified in Figure 3 that peak 2 at 9.76 min had significantly higher response than the peak 1 at 9.13 min. Within a certain concentration range, the area of peak 2 was changed accordingly with different sulfite concentration. Figure 4 showed the LC‐MS results of the sulfite derivative mixture. The retention time (Rt) of 7.0 min peak was ascertained as the compound of RS^−^ (m/z 155.0), the Rt of 8.3 min peak was assigned to the compound of RSSO_3_^−^ (m/z 235.5), and the peak with a Rt of 29.5 min and a molecular weight of 187 was supposed to the ionic fragment of DTNP (RSS^−^). It was also confirmed that SO_3_^2−^ and DTNP were reacted at a molar ratio of 1:1, as shown in Figure 1. Figure 3 also compared the HPLC chromatographs of standard sulfite solution, shrimp sample without added sulfites, and shrimp sample added with sulfites. The results revealed that no other compound in the sample could interfere with the derivatization process.

### 3.3 Calibration curves, precision, detection limits, and repeatabilities

**Figure 5:**
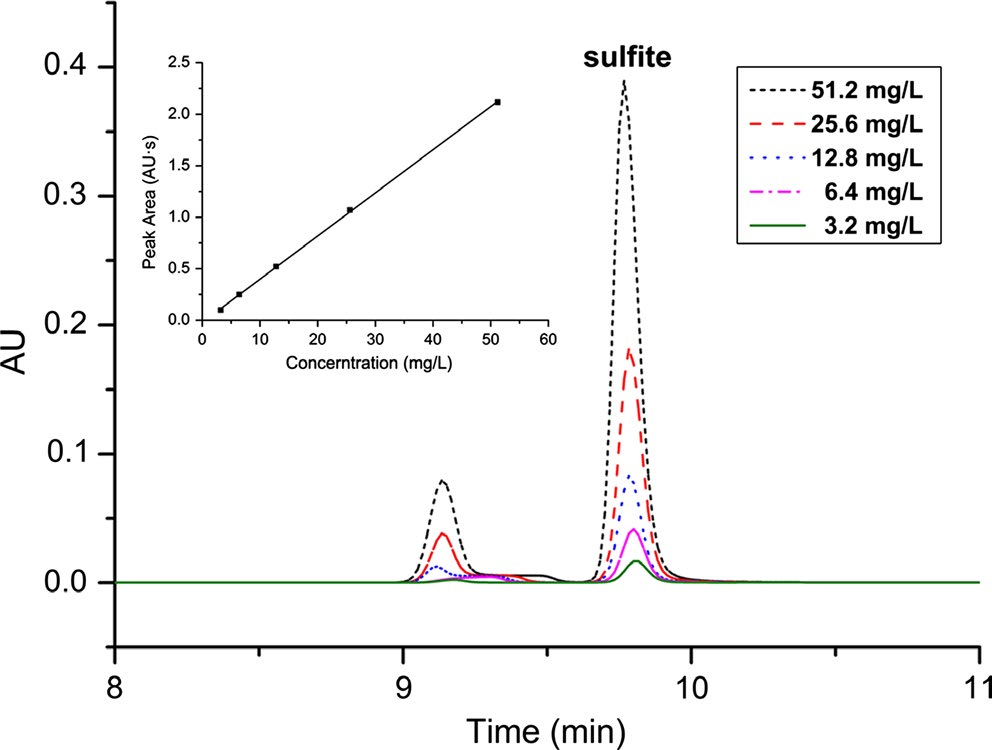
The calibration curves of sulfite derivatives (RSSO3^−^) determined by PD‐HPLC. PD‐HPLC, precolumn DTNP derivatization HPLC

Figure 5 presented standard curves of sulfite at a concentration range of 3.2–51.2 mg/L, dissolved in 0.02 mol/L of acetic acid–sodium acetate solution (pH 6.8). The inserted figure depicted the sulfite correlation curves, calculated as the corresponding regression equation of full name (PA) (Response value, AU·S) = 0.04192 × *C*‐0.02039 (C: concentration of SO_2_, mg/L), with an excellent correlation coefficient (*R* ^2^ = 0.9996). It could be seen in Table 1, the intraday precision was better than the daytime precision. The intraday precision and interday precision of the total sulfites under alkaline extraction conditions were slightly lower than the free sulfites, which might result from the effect of higher pH on the stability of the derivative product. However, the RSD values were all within 4%, meeting the testing requirements. Moreover, the limit of detection (LOD) and limit of quantitation (LOQ) of sulfites were 0.3 and 1.0 mg/L, calculated as SO_2_, based on a signal‐to‐noise ratio of 3 (S/N = 3) and 10 (S/N = 10), respectively.

**Table 1:** Result of precision test

Factor	pH	Standard sulfite solution				Shrimp samples			
		Intraday precision RSD (%)		Interday precision RSD (%)		Intraday precision RSD (%)		Interday precision RSD (%)	
		Rt (min)	Area	Rt (min)	Area	Rt (min)	Area	Rt (min)	Area
SO_3_^2−^	8	0.38	0.97	0.89	1.02	0.43	1.28	0.62	2.34
	12	/	/	/	/	0.62	1.56	0.98	2.81

Note

The free sulfite content in the standard solution is the same as the total sulfite content.

### 3.4 Sample analysis and comparison

Three different levels of standard sulfites (5.0, 20, and 50 mg/kg, in content of SO_2_) were added into the frozen shrimp homogenates. The precision and recovery rates of the PD‐HPLC method were carried out instantly, and the parallels were also conducted and compared with an IC method. As described previously, the free and total sulfites were selectively extracted from the matrix at pH 8.0 for free sulfites and pH 12.0 for total sulfites. Both of them were determined by PD‐HPLC and IC methods, and all the results were listed in Table 2. The recovery rates and relative standard deviations (RSD) obtained by PD‐HPLC method were 92.6%, 1.4% (5.0 mg/kg), 89.2%, 2.0% (20 mg/kg), and 94.8%, 1.2% (50 mg/kg), respectively. In terms of IC method, the recovery rates and RSD were 87.2%, 2.9% (5.0 mg/kg), 89.8%, 2.5% (20 mg/kg), and 85.2%, 1.8% (50 mg/kg). In addition, Table 2 also confirmed that the free sulfites were the main form of sulfites in shrimp, and the ratios of free sulfites to total sulfites were 0.85 and 0.89. Moreover, the PD‐HPLC method had better recoveries rate than that of IC method. Moreover, the developed method has achieved an improvement in the sensitivity compared to the previous work, such as chemistry, electrochemistry, spectrophotometry, IC, and GC (Aberl & Coelhan, 2013; Soares et al., 2013; Wang & Xu, 2014; Zhong et al., 2012; Zuo & Chen, 2003). LODs (0.3 mg/L) obtained in this study were better than or comparable to those of the reported detection methods. HPLC is also favored as a relatively common and easy‐to‐use instrument nowadays, in comparison with IC and cyclic voltammetric electrode.

**Table 2:** Comparison of the methods of PD‐HPLC and IC with recoveries and RSD of sulfites in shrimps (*n* = 3)

Standard sulfites added level (mg/kg)^a^	Sulfite contents determined in the shrimps (mg/kg)							
	PD‐HPLC		Recovery (%)^b^	RSD (%)^c^	IC		Recovery (%)^b^	RSD (%)^c^
	Free SO_2_	Total SO_2_			Free SO_2_	Total SO_2_		
0	26.58 ± 0.48	31.44 ± 0.83^A^	/	/	26.88 ± 0.69	32.73 ± 0.85^A^	/	/
5	31.09 ± 0.75	36.07 ± 0.86^B^	92.6	1.4	30.97 ± 1.13	37.09 ± 1.09^B^	87.2	2.9
20	43.63 ± 1.12	49.28 ± 1.01^C^	89.2	2.0	43.85 ± 0.93	50.68 ± 1.28^C^	89.8	2.5
50	70.72 ± 1.43	78.85 ± 0.69^D^	94.8	1.2	71.30 ± 1.06	75.34 ± 0.77^D^	85.2	1.8

Note

^A,B,C,D^Means with different letters within the same row (total SO_2_) are not significantly different (*p* > 0.05).

a Expressed as mg/kg of SO_2_.

b Recovery calculated as the determined total sulfite divided by added level.

c RSD calculated as standard deviation (*SD*) divided by total SO_2_.

## 4 CONCLUSIONS

In summary, a HPLC involving precolumn derivatization method for the analysis of sulfites in shrimps has been developed. The derivatization was based on the reaction of SO_3_^2−^ with DNTP and verified by LC‐MS. UV and visible absorption spectrum showed that all the derivatized products had absorption at 320 nm, and they were well separated by gradient elution in HPLC. The linearity was good in a range of 3.2–51.2 mg/L (*R* ^2^ = 0.9996). The proposed method could be successfully used to detect sulfites in shrimp samples with high recoveries (89.2%–94.8%) and reasonable relative standard deviations (RSD <2.4%). The detection limits of the PD‐HPLC method were slightly lower than the IC method. Accordingly, this new developed method can be used to determine the residue of sulfites in shrimp and other foodstuffs.

## CONFLICT OF INTEREST

The authors declare no conflict of interest.

## AUTHOR CONTRIBUTIONS

Authors K. Yang led the relevant project, designed the study, interpreted the results, and revised the paper. C. Zhou and Z. Yang designed and carried out the experiments, performed the data analyses, and wrote the manuscript draft. L. Yu provided the shrimps and collected the test data. C. Wu, M. Cai, and P. Sun helped with data analysis, polished the language, and edited the manuscript.

## ETHICAL STATEMENT

This study does not involve any human nor animal testing.
